# The continuum of gender-based violence experienced by migrant and refugee women in Canada: perspectives from key informants

**DOI:** 10.3389/fsoc.2024.1420124

**Published:** 2024-10-10

**Authors:** Mia Sisic, Evangelia Tastsoglou, Myrna Dawson, Catherine Holtmann, Lori Wilkinson, Chantelle Falconer

**Affiliations:** ^1^Department of Psychology, University of Windsor, Windsor, ON, Canada; ^2^Department of Sociology and Global Development Studies, Saint Mary's University, Halifax Regional Municipality, NS, Canada; ^3^Department of Sociology & Anthropology, University of Guelph, Guelph, ON, Canada; ^4^Department of Sociology, University of New Brunswick Saint John, Saint John, NB, Canada; ^5^Department of Sociology and Criminology, University of Manitoba, Winnipeg, MB, Canada; ^6^Faculty of Management, Dalhousie University, Halifax, NS, Canada

**Keywords:** gender-based violence (GBV), women, migrants, refugees, key informants (KIs)

## Abstract

Little research has been done on conceptualizing gender-based violence (GBV) against immigrant and refugee women as a continuum of violence. The objective of the larger study was to understand gender-based violence in migration and analyze the ways in which discriminations and inequalities interact to increase vulnerability and decrease access to supports and services for some women. Using (a) the concept of continuum of [sexual] violence and (b) intersectionality, we demonstrate the need to both document the range of violence in women’s lives and the tactics of victimization among immigrant and refugee women and show how they are different than the cumulated literature showing victimization tactics against the Canadian-born population. Using semi-structured interviews via phone or video, we asked professionals (*N* = 43) who worked with migrant women across Canada about forms of GBV experienced in the immigrant and refugee populations they worked with. Participants reported that non-physical forms of violence are more normalized, but also more commonly experienced than physical forms of violence in Canada. Additionally, intersecting social identities impact both the distinct and amplified forms of GBV immigrant and refugee women experienced. Results contribute theoretically and empirically to the conceptualization of the GBV experiences by immigrant and refugee women in Canada.

## Introduction

Nearly one in four women and girls living in Canada are current or former newcomers (Statistics Canada, 2023) and the proportion is expected to rise (Hudon, 2015). Although the number of asylum/refugee claimants can vary greatly by year, approximately 9,000 women (48% of total applicants) were principal applicants in an asylum claim in 2019 (Government of Canada, 2021). One of the reasons that migrant and refugee women often come to Canada is to escape previous trauma or ongoing gender-based violence. Although varying forms of gender-based violence/persecution are common reasons cited by refugee claimants, not all women making such claims are successful in gaining asylum (Carman and Elash, 2018; Tastsoglou and Nourpanah, 2019). Among women refugee claimants, commonly cited reasons for seeking asylum include domestic violence, forced marriage, and female genital mutilation (Carman and Elash, 2018). We use the terminology of “migrant and refugee” to denote the broadest possible range of different legal statuses of migrant arrivals in Canada (e.g., immigrants, temporary migrants, convention refugees, refugee claimants, and individuals without legal status). While migration status plays a major role in the specific rights and entitlements of migrants, we focus here on the general, common characteristics of their GBV experience.

This article derives from a larger study the objective of which was to understand gender-based violence in migration and analyze the ways in which discriminations and inequalities interact to increase vulnerability and decrease access to supports and services for some women. This paper focuses on Key Informants’ understandings of the experiences of gender-based violence (GBV) against migrant and refugee women in Canada (MRW). For our study purposes, Key Informants are individuals who provide professional services and support to such women. As professionals in the settlement and anti-violence sectors, Key Informants play a crucial role in shaping policy and programming for immigrants, migrants and refugees when they first arrive in Canada and during their first few years of settlement. Drawing upon Kelly’s (1987) theory on the continuum of sexual violence, we apply a much-needed intersectionality lens to the issue of GBV experienced by migrant and refugee women, as perceived by Key Informants. Some of that violence has preceded the women’s arrival to Canada and constituted the reason for their migration; some occurred during long and arduous journeys before they arrived in Canada; and some took place in Canada. Most importantly, however, the “continuum” refers not just to ongoing journeys but to qualitative continuities and linkages of GBV forms, across space and time. The intersectionality framework sheds light on the significance of this continuum in shaping the experiences of migrant and refugee women, in the context of differential, but always higher vulnerabilities that migration and refugee journeys entail.

Starting with a critical overview of GBV forms in Canada, we proceed to draw upon Kelly’s (1987) theory and the intersectionality approach in order to understand the migrant and refugee women’s GBV experiences and the role that intersectionality plays in such experiences for the survivors from the perspectives of Key Informants from across Canada (individuals who work to prevent GBV and/or support those victimized by GBV). More specifically, in our research: (a) we *contract* Kelly’s (1987) continuum theory by highlighting the experiences of a particular population, namely migrant and refugee women; (b) at the same time focusing on this particular population intersectionally, allows us to *expand* Kelly’s continuum theory by adding unique forms of GBV or unique risk factors systematically amplifying vulnerabilities of MRW. We claim that this naming of new GBV forms is significant and cannot be ignored by lumping them all together as GBV; and, finally, (c) we discuss the implications of our analysis for policy needed to more effectively address GBV.

### Forms of gender-based violence against migrant and refugee women in Canada: an overview

Migrant and refugee women residing in Canada are victimized by a variety of forms of GBV; however, in academic literature, most of the research focuses on intimate partner violence (IPV). Racialized minority women have lower rates of IPV compared to Canadian-born women, but this could be because migrant women may be more reluctant than Canadian-born women to disclose IPV to interviewers due to language or cultural barriers (Brennan, 2011; Brownridge and Halli, 2003; Cotter, 2021a). Namely, migrant women may face unique risk factors for and vulnerabilities to IPV due to structural inequalities at the intersection of migrant identity, gender, ethnicity, and class, as well as intersections between structural and cultural factors acting as impediments to seeking help (Abraham and Tastsoglou, 2016).

Forced marriages and femicide are other forms of GBV that less is known about, and particularly about the experiences MRW face. While an estimated 70 cases of forced marriages per year have been documented in Ontario, with 92% of those victimized being women, forced marriages are not restricted to citizenship status, particular geographic area or culture (Anis et al., 2013). The number of cases of GBV does not necessarily include arranged marriages and this may be an important distinction to consider because arranged and forced marriages “shade into one another through varying degrees of social and cultural expectation” (Anitha and Gill, 2017, p. 180). As for femicide, the proportion involving migrant victims is not known, and nationwide data are not available; however, at least 99 migrants and refugees were victims of domestic homicide between 2010 and 2018, 87% of which were women (Dawson et al., 2018). Further, though prevalence of “honor-killings” is difficult to determine, we know that it does occur: there were an estimated 10–15 cases in Canada from 2002 to 2012 (Korteweg and Yurdakul, 2012).

Human trafficking is of increasing concern (Kaye et al., 2014), but also with limited data, due partially to the hidden nature of the crime, and because victims tend to distrust police, experience language barriers, or are unaware of their legal rights (Ibrahim, 2018). Instances of police-reported human trafficking have been increasing since 2010 with 95% of those victimized being women, many of whom were young (Ibrahim, 2018). Women who experience multiple forms of oppression combined with their sex or gender may be at higher risk of being trafficked (Kaye et al., 2014).

There are limited data available on female genital mutilation, but it is expected that girls in Canada are not safe from the practice. It is also difficult to estimate the number of people seeking asylum claims on the basis of lesbian, gay, bisexual, trans, or queer (LGBTQ) identities (Fox et al., 2020). However, the combination of anti-LGBTQIA+ discrimination (e.g., homophobia, transphobia) and misogyny makes queer and trans women vulnerable to specific gendered forms of violence (e.g., “corrective rape”), particularly in combination with class, immigration status, and race-based oppression (Dearham, 2017).

### The continuum of sexual violence

The continuum of sexual violence, a concept developed by Kelly (1987), describes the range and extent of sexual violence women face based on interviews with women and their experiences with sexual violence, including the more hidden aspects of violence. Recently, Kelly (2012) has clarified that the term “sexual violence” at the point of development of the continuum (and on) was a term encompassing all forms of violence against women and is equivalent to more contemporary terms “violence against women” and “gender-based violence.” This paper takes and supports that position. In the original study, women were asked about a range of experiences and a tally of the reported incidences of violence was recorded. The incidences were then organized from the most to the least common forms of sexual violence and represented on a continuum. In the current study, we asked Key Informants which forms of GBV, in their opinion, are most pressing. The question posed to the Key Informants was used as a proxy for reported incidences of sexual violence in Kelly’s study with women.

The continuum has two meanings: (a) there is a basic common character to the sexual/gender-based violence (i.e., men use different forms of abuse, coercion, and force to control women) and (b) there are no distinct categories of sexual/gender-based violence, because they all “pass” into one another. Both meanings highlight that most women have been victimized by sexual violence in some way, although the forms of violence, how women define the violence, and the impact of violence varies.

As one moves through the continuum, less frequent forms of sexual/gender-based violence are encountered (e.g., incidences of sexual harassment are more common than incidences of incest). Namely, the continuum is not about seriousness or severity of effects on the women (unless it is death^1^) because it is not a hierarchy of sexual violence. The forms of violence that most women experience, and on more occasions (i.e., common forms of violence), are less likely to be criminalized in law and are also the ones that men are more likely to define as acceptable behavior (e.g., sexual harassment conceptualized by men as a “joke”).

The continuum as a concept according to Kelly’s theory can be used in three main ways. First, the concept captures everyday forms of violence (e.g., threats) without focusing on extremes of violence such as physical force. Second, it links aspects or forms of men’s violence against women with common interactions between men and women. Finally, women can use the continuum to link typical (i.e., everyday male behavior) and atypical male behavior which enables women to locate and name their own experiences of sexual violence.

The continuum has been used to understand different forms of men’s violence against women such as the relationship between financial abuse and other forms of violence against women (Eriksson and Ulmestig, 2021), and recognizing and predicting abuse escalation (Barata et al., 2005). In a forced migration context, Kelly’s continuum of sexual violence has also been used to argue that different forms of GBV connect and overlap throughout conflict, flight and displacement (Krause, 2015; Tastsoglou, 2022). More recently, the continuum concept has been applied beyond the refugee journey into re-settlement, with asylum-seeking women being victimized in accommodation facilities and through inadequate service provision in receiving societies (Sullivan et al., 2021; Phillimore et al., 2022; Sahraoui and Freedman, 2022).

Key Informants drew on MRW experiences within the Canadian context (i.e., violence that began after settlement in Canada or *continued* from the country of origin). In that way, the results capture violence across time and geographic location, though primarily rooted in Canada. Starting from structural violence as a backdrop for the victimization of women by men we include more types of violence (and more people/groups) in Kelly’s continuum of sexual violence. There are unique forms of GBV and GBV vulnerability in a Canadian migration/refugee context that emerge only upon understanding the intersectional discriminations framing migrant and refugee women’s lives. Understanding them helps assess how these forms may shift the range of the GBV continuum. Such understanding does not detract from the universal problem (GBV and its continuum of forms); on the contrary, it recognizes the particular forms of GBV thereby enabling targeted policy responses. Feminists have discussed the importance of “differentiated universalism” (Lister, 1998) for the feminist movement and the need for “transversal dialogue” (Werbner and Yuval-Davis, 1999).

### Intersectionality

The point of departure of a feminist intersectional perspective is that women and men do not form essentialized, hermetically sealed, homogenous groups, with similar experiences and identities within. On the contrary, they are differentiated by diverse power relations, unequal social positions, and identifications built thereupon (Tastsoglou, 2019). Gender power relations, which typically disadvantage women and gender minorities, persist in all societies and interweave with other forms of social divisions to distribute power and resources in context-specific ways. Gender inequalities, intersecting with other hierarchies of power are embedded in the organization of social institutions and manifest themselves on the level of interpersonal relations, experiences and identities. Feminist theorists such as Choo and Ferree (2010), and Hill Collins (2010) have argued that these power relations, social positions, and identifications are mapped on a broader and shifting matrix of intersecting structural inequalities and oppressions within a global capitalist context.

Intersectionality has a rich history with antecedents in United States and British Black feminist thought (Reilly et al., 2022). The term itself was coined by United States feminist and anti-racist legal scholar Kimberlé Crenshaw (1989) in her seminal law review article that pointed out the necessity of demolishing a “single-axis analysis” (Tastsoglou, 2019). Intersectionality was diffused globally and became very influential not only in feminist and anti-racist theorizing but also in government and United Nations’ discourse. A rich and ever-growing literature has understood intersectionality in a variety of often complementary ways: For example, “a matrix of domination” consisting of intersecting axes of oppression (Hill Collins, 1990, 2010); a multi-level historical co-determination of interactive racialization, gendering and class forming processes (Choo and Ferree, 2010); a “knowledge project” situated within the power relations it studies (Hill Collins, 2015); a “work in progress” inviting researchers and activists to always broaden the scope of work where intersectionality can be mobilized (Carbado et al., 2013; Marfelt, 2016); an “activist” approach animated by the motive of social change and social justice (Hill Collins, 2015). In our own analysis, the intersectionality approach leads us to identify the specific impacts in the experiences of GBV of intersecting social positions and identities built upon women who carry the legal and social statuses of migrants and refugees in Canada.

Intersectional frameworks have often been employed in research examining MRW’s experiences of GBV. For example, particular heightened stressors (e.g., social isolation, language barriers) have been identified, and viewed as associated with their immigration status intersecting with other social identities, such as those based on race and class, to exacerbate the experiences of GBV and prevent women from challenging the abuse (Erez and Harper, 2018). However, only limited research has examined how forms of GBV may qualitatively differ for MRW (e.g., men threatening to report wives to immigration officials; Erez et al., 2009), their frequencies of occurrence, and how, as a result, the continuum of violence involves a different range of GBV. It is those differences in the experience of the continuum of GBV that necessitate that we focus on understanding the GBV continuum specifically for MRW in Canada, without any assumptions and projections based on the continuum of sexual violence for the general population of women. The present article focuses on the specificities of GBV experience for MRW as such specificities are perceived by Key Informants who work with migrants and refugees. The article’s findings focus on the intersection of gender and migrant/refugee status, as our data regarding other identities (e.g., race, religion) is too limited to make any broader claims.

## Methodological considerations

This paper draws upon data from the Canadian research program,^2^ associated with the international project *Violence Against Women Migrants and Refugees: Analyzing Causes and Effective Policy Response*. The data analyzed in this paper are derived from interviews with 43 Key Informants from across Canada, conducted between fall 2019 and summer 2020.

Key Informants included professionals who worked either directly or indirectly with migrant women. They were most often employed in either the settlement or anti-violence sectors and had a role in developing policy and programs or delivering supports to migrant women who had experienced GBV. Approximately one third [15] of the Key Informants were service providers in the immigrant settlement sector (e.g., counselors, administrators) and another third [13] worked with domestic violence survivors in varying capacities (e.g., counselors). Six Key Informants were government workers who worked on immigration, policy, and women’s issues [6] and five were NGO members. Two Key Informants worked in legal services for refugees and immigrants; two worked in health care clinics for refugees (for more detail, please see Holtmann et al., 2023). A pan-Canadian Expert Advisory Group advised on key issues related to this work, such as recruiting and interviewing with trauma-informed approaches to research.

Our semi-structured interviews used an interview guide developed in collaboration with the international project teams. The guide was tailored to the Canadian context, with questions covering a range of themes including types of service users, forms of violence, services offered, work challenges and ideas about improvement. We fully acknowledge the indirect source of our information about the GBV experiences of MRW as well as the fact that Key Informants did not speak with one voice about GBV but from their own professional and social positions with all the insights and shortcomings associated with them (Oliveira et al., 2019). In this paper, we focus our analysis on the forms of GBV they collectively identified from their perspectives.

Interviews were completed remotely by three interviewers, either over the phone or videoconference. The decision for remote interviews was partially to facilitate recruitment of participants from varied geographic locations, but also because most of the interviews were conducted during travel restrictions limiting in-person meetings due to COVID-19. Interviews varied in length from approximately 30–150 min. All interviews were conducted in English except one which was conducted in French. Interviews were audio recorded, transcribed, and thematically analyzed (Braun and Clarke, 2006). This paper focuses on two themes that were generated from this analysis: (1) forms of GBV and (2) intersectional processes shaping experiences of GBV. All participants discussed a range of forms of violence by which women were victimized. Many participants talked about non-physical forms of violence (e.g., financial, psychological) being the most pressing forms of GBV. Some participants also spoke about why non-physical forms of violence are pressing forms of GBV such as their higher frequency, higher likelihood to be perceived as ambiguous, and lower likelihood to be taken seriously.

The perspectives of Key Informants and how they understand and recognize GBV are particularly important because (a) they are in a position to recognize red flags; (b) they are in proximity to migrants and refugees, given that the latter do not have their familiar social contexts and supports in Canada; (c) professionals are aware of the gaps in migrant and refugee services and able to identify them for us as researchers—they are aware of vulnerabilities that cross-cut different groups. At the same time, the major drawback of accessing information through Key Informant voices has to do with their perspectives, interpretations and priorities not necessarily coinciding with those of MRW (Lokot, 2021). Despite this shortcoming, speaking with Key Informants was part of our trauma-informed approach to data collection: by first gathering insights from those who work in close proximity with MRW. We were sensitive of the burden MRW may feel having to retell their stories in order to access services, legal status, etc.

## Findings and discussion

Our findings in this section are grouped under two broad categories. In the first, drawing upon Kelly’s continuum theory we examine the commonalities between Key Informants’ perceived experiences of violence against MRW with that of existing literature on Canadian-born women; while, in the second, we assess how MRW’s experiences of GBV stand apart from those of Canadian-born women using the aforementioned literature.

### Commonalities in the continuum of sexual/gender-based violence

#### Forms of GBV

Unlike Kelly’s approach, we did not ask about frequency of violence the Key Informants’ clients experienced. However, we did ask the participants what they felt were the most pressing forms of violence (i.e., subtheme: “most pressing forms of GBV” which was coded 80 times). Non-physical forms of violence accounted for most of the answers and included emotional (*n* = 9), control and manipulation (*n* = 7), psychological (*n* = 7), financial (*n* = 6), and verbal (*n* = 2). For two forms of violence, sexual violence (*n* = 9) and IPV (*n* = 5), it was unclear whether these responses were about physical or non-physical forms of violence. For example, IPV is widely known to include both physical and non-physical forms of violence (e.g., Cotter, 2021b). Physical violence (*n* = 7) was also cited. See Table 1 for a complete list. The number of times each answer was given was somewhat irrelevant to the overall analysis, but is used to show “links between the different forms of sexual violence” (p.46), regardless of location. The violence that the Key Informants most often reported on was the violence they were privy to in the Canadian context: violence that either continued from the refugee or migrant woman’s home country or began in Canada.

**Table 1:** Frequency of citing for “most pressing forms of gender-based violence” subtheme.

Form of violence	Frequency of answer	Clarification
Sexual	9	
Emotional	9	
Other kind of violence	8	Most answers focused on population such as children, girls, or transgender, but not the *form* of violence.
Physical	7	
Psychological	7	
Control and Manipulation	7	
Undecided	7	Unsure; inability to rank, perception they are all pressing
Financial	6	
Intimate partner violence	5	
Unclear	4	
Inter-related violence	3	Forms/types of violence cannot be separated; they are all related and co-occur
System/structural	2	
Verbal	2	
General non-physical violence	2	

In this section, we argue that the results of the first theme with respect to MRW are aligned with Kelly’s (1987) general overview of sexual/gender-based violence. In sum, like Canadian-born women, (a) MRW are more commonly victimized by non-physical forms of violence rather than physical forms of violence; (b) non-physical and physical forms of violence are interlinked, but non-physical forms of GBV are normalized, delegitimized, and rendered invisibile in Canadian society underscoring the need to conscientiously expand conceptualizations of GBV beyond physical violence.

#### Focusing on everyday/common forms of violence

Kelly’s concept of sexual violence as a continuum has several important features used as an organizing principle in these results. First, the continuum refers to incidences of abuse and should not be interpreted as a continuum of seriousness or how severe the effects of abuse can be on a woman. From the participants who answered that physical violence was the most pressing form of GBV (interview question: In your opinion, which forms of GBV are most pressing?), several noted the caveat that non-physical forms of violence were just as pressing. KI16^3^ explained that although physical violence is the most pressing form of GBV, non-physical forms of violence happen more often than physical violence.

[Most pressing form of gender-based violence] could be physical, of course it’s physical abuse, it’s physical violence, it’s threatening, it’s threat to use physical violence or some force. And of course, it’s when the power balance is vulnerable and weak (…) For example, for sponsored immigrants, sponsored spouses, a lot of psychological abuse, a lot of emotional abuse, threatening and actually when the victim is not touched physically but she’s worked out to exhaustion by this attitude, relationships or this treatment. We deal a lot with these issues. I do not think it’s on the rise, but it was always a very high percentage. It’s a little bit higher than physical violence.^4^

Participants spoke about serious consequences of non-physical violence (e.g., victim suicide attempts) noting that victimized women often told them that non-physical forms of violence were more difficult to heal from, in part because physical violence is easier to comprehend as violence:

KI21: Many women they tell me emotional and psychological abuse are more difficult to see and then to heal from. You know if you experience physical abuse. You know. You can see and you can understand and you know, it’s not right.

Women are more likely to contact the police if they are victimized by physical forms of violence (e.g., sustained physical injuries; Akers and Kaukinen, 2009), suggesting either that they, or others (e.g., service providers the women contact), are more likely to recognize them as violence. Although victimized women downplay physical forms of violence (Dunham and Senn, 2000), they may be more likely to downplay non-physical forms of violence (e.g., emotional abuse) though they can have serious consequences (e.g., depression; Matheson et al., 2015). Non-physical forms of violence are indeed far more common (Cotter, 2021b) even in cases when physical abuse is present (e.g., Sullivan et al., 2012). Thus, it is important to expand research and everyday discourses to include non-physical forms of violence, at least in part because they are more common and can have deleterious consequences (Matheson et al., 2015). In lieu of the frequency of forms of SV in Kelly’s continuum, here our findings refer to the “most pressing forms of GBV,” raised by KIs, as being indirect, invisible, and normalized. Additionally, the range of violence for immigrant and refugee women may be different than for Canadian-born women and include unique experiences like instances when the perpetrator controls the women’s access to language classes or language acquisition which, in turn, makes it difficult to seek support services or access information about the law.

#### Linking the forms of men’s violence against migrant and refugee women

Another feature of the continuum is that men’s violent acts against women and girls are linked by a common underlying factor: men use varying methods of abuse, coercion, and force to control women. For example, the majority of women experience multiple forms of violence (Thompson et al., 2006), of which non-physical forms of violence (e.g., stalking) may be more likely to be accompanied by other forms of violence (e.g., physical, sexual; Krebs et al., 2011), may last for longer periods of time (e.g., controlling behavior; Thompson et al., 2006) and are linked to physical violence later on in an intimate relationship (i.e., “escalation of violence”; Murphy and O’Leary, 1989). These features of violence were reflected in interviews with Key Informants regarding the victimization of MRW: one form of violence can escalate to the point of physical violence while encompassing other forms of violence:

KI35: As usual, there is a combination, it’s not one or another. (…) I mean, this escalates. At the beginning, it can start by certain controlling behaviors and at the end it will entail **also** [added for emphasis] physical violence. And the psychological violence, the emotional violence is there. So [it] is not one or another. Many of these different fragmented ways to violence will be combined. It’s like a constellation. We cannot just tease out one.

Participants spoke about the normalization of the most frequent kinds of GBV which is a pressing issue. KI4 added that the normalization means that some forms of GBV are not flagged as violence and do not receive the same response from society:

We flag certain things as violent right. But all of that kind of normalized [violence] (…) do not get the same response from our society (…) For me it’s that kind of normalizing practice that happens, that to me is the most pressing.

This normalization can occur in two ways: First, some participants noted that non-physical forms of violence are “culturally” sanctioned forms of behavior and normalized:

KI12: I’d say from a newcomer clinic perspective, from the patients that I see it is the emotional and financial violence that is subtle and maybe culturally sanctioned in other…well even here frankly, but can be culturally sanctioned but is insidious for a lot of the women I see.

By accepting the notion of “culturally sanctioned violence” some participants reproduced societal stereotypes about violent cultures (which are perceived to practice violence that is not known or characteristic of/in Canadian society), but also illustrated how dismissive society is of certain forms of violence as “cultural.” Additionally, however, participants understood Canadian culture as being implicit in the normalization of violence. KI32 gave the example of some types of violence being framed as “fights,” an acceptable notion in Canadian society: “Society in general is like ‘everybody gets in fights’ but it’s more than that.”

Second, participants implicated society more broadly in the normalization of non-physical forms of violence, including specific systems such as media and the criminal justice system. They viewed these systems as reproducing society’s differential treatment of physical and non-physical violence (e.g., focusing on physical violence). KI27 described a situation in which a woman pressed charges against her male partner for physical abuse. After his release, he used non-physical forms of violence as he became aware that he would not be criminally charged for those:

She did press charges last year, okay. And since then, he’s very well aware that if he should threaten her or use any type of physical force that he will be probably jailed and, who knows in his situation perhaps, sent back because she sponsored him. (…) But so now what happens is that he can continue to be psychologically and verbally abusive toward her, ignore her, be very indifferent, tell her she’s not a good mother. But that’s not criminal charges. That does not warrant a criminal charge so he knows exactly just where not to cross the line.

The framing of some forms of violence as more legitimate or more socially and legally recognized (most commonly these are the most explicit and extreme forms of violence) than others and the non-recognition or invisibilization of certain forms also has an impact on responses to GBV, beyond the criminal justice system and reaching into policy. KI4 explained that physical forms of violence are legitimatized forms of violence – at the expense of other forms being delegitimized:

We identify these are like physical forms of violence. This is the way it’s legally sanctioned right. I’d call that kind of more legitimate forms of violence [and] (…) the limitations of framing it in that way right (…) trying to understand the way that it’s being thought of, the way that it’s in practice, the way that it gets legitimized but then all of the other forms of gender-based violence just seem to not be able to be labeled or identified right. (…) Because the way we are thinking about it and the way we talk about it then in turn becomes the way that we respond to it. And if we do not even have the capacity, the literacy, let us say, to look at certain forms as violent right. Then there’s no way that there’s a policy around it.

The perception of what constitutes men’s violence against women varies in the law, across states and times. For example, feminist activists and researchers have fought for decades, with some success, for broadened definitions of men’s violence against women and in opposition to dominant Western definitions of violence which often focus on physical violence (e.g., DeKeseredy and Schwartz, 2001; Senn, 2000). Conceptualizations and definitions of what constitutes GBV need to be broadened to include non-physical forms of violence with the understanding that while these forms of violence are likely to be minimized or labeled as a joke (Lockyer and Savigny, 2020) and less likely to be taken seriously by the criminal justice systems (Powell and Henry, 2018), broad definitions of GBV that include structural arrangements in (re)producing GBV may trivialize the experience of violence for including “everything but the kitchen sink” (e.g., DeKeseredy and Schwartz, 2001). As such, social scientists and others should be urged to consider “who benefits, who loses, and what is implied” (p.243) through the questions asked about GBV and how GBV is defined (e.g., recognize the benefits and limitations of framing violence only as extreme physical force; Muehlenhard and Kimes, 1999).

### An amended continuum: an intersectional approach to GBV experiences of migrant and refugee women in Canada

In this section, we explore the intersections of social identities (e.g., gender, legal status) on the GBV experiences of MRW with a focus on forms of GBV either specific to MRW or aggravated by their migrant/refugee identity. Discrimination based on an intersection of gender with other social identities was a finding that echoed throughout our interviews. This discrimination was tied to the stereotypical and narrow idea of what violence looks like. KI25 explained that the experiences of most people, particularly those who are marginalized in some way (like women who do not have status) are not readily reflected in public conversations about violence:

We’re still stuck on a really kind of stereotypical idea of what violence looks like (…) When people hear gender-based violence, they think mostly of domestic violence, as in physical violence, or sexual violence (…) that really erases the experiences of most people actually. And then particularly the experiences that we would see non-status women experiencing or other women with disabilities, other populations who have the highest rates of gender-based violence, but whose experiences aren’t always being reflected in sort of the public conversation around it.

Drawing from our interviews, we identified five pathways—constituting risk factors—in which a woman’s migrant or refugee status impacted the specific types of violence she experienced. The first three pathways—social isolation, threats of child apprehension, and involvement of extended family—may be common to many, non-migrant women impacted by IPV as well. We argue that the first three pathways/risk factors produced much amplified vulnerabilities for MRW. The second two pathways/risk factors—language barriers and legal status—signaled vulnerabilities to GBV distinct to MRW. For example, women from countries of origin where French or English is not an official language (or when one of the two languages is not spoken by the MRW) would experience language barriers as a risk factor producing vulnerability to violence that Canadian-born would likely not.

#### Social isolation

Social isolation was perceived to be particularly problematic among MRW as well as a form of violence in and of itself. For some women, the migration to Canada was used as strategic abuse to isolate the woman from her family; for others, the abusive partner uses social isolation to gain or maintain control over the woman (e.g., she is escorted to all public spaces to keep her from gaining any connections). KI29 emphasized the role of digital space in social isolation:

So there might just be one laptop in the home. Who has use of a laptop? Who has the literacy to use the laptop? Who prevents whom from attending digital literacy classes? Who controls the Wi-Fi passwords? Who limits the amount of time that a person is online because if you can isolate them, you can isolate them every which way not just physically but also electronically (…) She cannot look up, she cannot book anything. She cannot go home. She cannot see her family. She cannot ask them to come over to visit her. She cannot Skype with them.

In some cases, the social isolation can be so extreme that a community may not know the woman even exists. KI34 recalled a woman who was sponsored by her husband who had controlled many aspects of her life, including her social interactions. He had kept her inside the home and isolated to the point that the organization did not even know that she had arrived in their community:

We had a woman that we did not even know. Basically, a former refugee who was now a citizen sponsored her as his wife and brought her to the country and her sponsor became her abuser. We did not even know she was here because she’d come through [a sponsorship] (…) he was her interpreter, he was her sponsor receiving the money, he was in charge of absolutely everything and she had not been out of the doors. She did not know the area, she knew nothing. Very isolated.

Social isolation is a common abuse tactic across a range of victimizations (Duron et al., 2021) and there is some evidence that perpetrators use isolation in IPV in specific ways depending on context and victim’s social identities (e.g., elderly women; Brandl, 2000). Though there are many theories on the relationship between GBV and social isolation (e.g., victim socially withdraws after abuse starts; Kim, 2019), our findings show that it is a perpetrator’s tactic of abuse and a form of violence in and of itself that can manifest as physical or digital social isolation against migrant women who have limited or no social networks due to leaving their family and friends behind when immigrating to Canada.

#### Threats of child apprehension

Some perpetrators use the possibility of children being taken away as a threat to keep the woman in the abusive home. KI42 explained that this is particularly difficult for migrant women who may not know their rights in Canada:

All sorts of threats like “You’ll never be able to survive without me. If you leave me, you’ll lose the kids, youth protection will take them.” That threat is particularly hard for migrant women who do not know their rights, they believe it.

KI8 added that, for some women, the kids being taken away may be a reality in their country of origin, so it is easier to believe that it will happen in Canada. IPV literature shows that husbands have threatened to take the children as part of IPV while in the relationship (Velonis et al., 2017) and post-separation (Toews and Bermea, 2017), although these threats typically involve the threat of filing for custody and kidnapping the children. These commonly used tactics were not often brought up by participants when describing threats made to MRW. Instead, men in relationships with MRW tend to deliberately use her lack of knowledge of the law, convincing her that Canadian law would work against her and leave her without her children (either in state or husband’s care). Additionally, when a perpetrator controls access to information/technology (above) and to language acquisition (see below), it may be impossible for a woman to know when she is being lied to about child apprehension. This not only demonstrates the exacerbated (non-physical) violence that MRW are victimized by, but also that the types of violence are not always distinct and may pass into another. This particular nuance of a common threat can be understood only with an intersectional approach and makes the threat unique for MRW.

#### Involvement of extended family

Threatening to use/using violence against the woman’s family in her country of origin was noted as an amplified and possibly more frequent form of GBV for MRW. The concern for one’s family being so far away and possibly existing generalized violence in the country of origin can make a threat of violence particularly effective in the case of MRW. KI32 cited an example of a client whose husband’s family killed somebody from the wife’s family to threaten the wife.

Other things that we see in relation to working with the immigrant and refugee population is using relatives back in the country of origin to threaten their partner’s relatives. We actually had somebody in Africa whose husband’s family killed somebody of the wife’s family to send a message. (…) We see a lot of that.

Existing IPV literature shows that animal (e.g., family pets) maltreatment is a form of violence committed against women (Fitzgerald et al., 2019) and that men’s violence against women extends to murder of their children (and suicide; Sev’er, 1997). It is unclear whether the situation is the same in IPV against MRW. Our data, however, suggest that threatening or using lethal violence against family members in another country is not atypical and that it may happen more frequently than in the general population of women victimized by IPV. Depending on the country of origin in question, the threat of resorting to violence against family members may appear more credible and terrifying pushing to compliance and submission of MRW.

#### Language barriers

Language barriers and controlling access to language acquisition are forms of violence used specifically against MRW. In addition, these forms of violence are pathways to increase vulnerabilities of MRW to further violence. KI27 described a situation wherein a woman she knew was trying to learn French, in part to better her education and to gain independence in Canada. Her partner, however, sabotaged her studying attempts:

She does not speak a lot of French (…) it’s difficult for her because now she’s just taken another course and will graduate but she has to pass a French course. (…) When she is studying or when she’s trying to study, her partner, because he does not really agree with her graduating and then gaining independence, will turn the TV up as loud as possible so she is not able to concentrate.

Language barriers are an often-cited impediment for MRW seeking help or services for GBV (e.g., Erez and Harper, 2018). Our results highlight especially how men control women’s language acquisition as a form of GBV, thus preventing women from gaining independence. Controlling access to language acquisition could also help account for why language continues to be a barrier for women trying to access GBV-related services (e.g., shelters, lawyers).

#### Legal status in Canada

Key Informants frequently spoke about legal status and its relationship to the specific forms of violence that MRW experienced. The violence can begin with refusal to sponsor and the intent to limit the woman’s rights (e.g., husbands refuse to sponsor, so wives are relegated to refugee status and, thus, limited rights in Canada). Once a woman is in Canada, her immigration paperwork can be withheld by the spouse to maintain power over her. This can be done in an effort to conceal her legal status in Canada from her:

KI8: It [GBV] also can be withholding of important immigration information. (…) That’s probably the big one. It’s the misinformation, the husband’s holding the passports and not telling them what their actual status is in Canada.

KI31 summed up comprehensively that women who do not have permanent residency or Canadian citizenship have their status in Canada abused by the perpetrators (e.g., threats of deportation, threats of job loss) as a form of control. The perpetrators also tend to be the ones who have a more secure legal status in Canada and prey on victims who have less secure status:

I think whenever a woman does not have permanent residency or Canadian citizenship, a lot of clients I’ve worked with fear that their immigration status will be affected if they do anything. So I find a lot of perpetrators will use that and abuse it to no end, like their immigration status like “if you call the cops, I’m gonna tell them this and this and you are gonna go back home” or “If you do this you are gonna lose your job” or “If you do this, the police are gonna do this.” They make lots of threats or feed my clients lies about police, about their immigration status, about all these different things to kinda keep hold of the control. I think those are huge factors in perpetuating that violence (…) I’ve worked with clients who the woman has a work permit or they even have permanent residency but maybe they have more of what we call a temporary work permit or something like that and the other person is a Canadian citizen and they use to sometimes prey on our clients.

Some Key Informants warned however, that spouses may play a limited role in deportation and that the myth of a partner being able to deport the woman needs to be addressed. Using legal status as a tool to victimize is unique to MRW that can be understood more wholly through an intersectional lens, taking into account specifically the legal and social status of the woman.

The effect of a woman’s legal status on her experiences of GBV has often been framed as a structural issue with a focus on women’s inaccessibility to important services (e.g., Côté et al., 2001). While we support this argument, men’s violence against women—in any form—is widely acknowledged to be a systemic (e.g., patriarchy) and structural issue (e.g., Abraham and Tastsoglou, 2016). This does not preclude holding abusers responsible for their abuse and focusing on specific forms of violence women are victimized by (in this case how legal status is used against them by their male partners). For example, many women do not leave abusive relationships because they (often correctly) fear that it is dangerous and that they may not get adequate support (a structural issue). In fact, separation is a key factor in intimate partner femicide (Dawson et al., 2021). In this section of the paper we show that MRW face amplified or different forms of GBV (e.g., legal status used as a form of violence) and argue that the definition of GBV needs to be expanded to include a wider variety of forms of GBV and particularly those that affect women at the intersection of gender and migrant/refugee status.

## Conclusion

This study has made some key contributions to the existing literature. First, we have showed empirically that the continuum of sexual violence (Kelly, 1987) applies as well to MRW victimized by GBV. Second, using an intersectional approach we have demonstrated that the continuum of sexual/gender-based violence for MRW comprises both similar (Jiwani, 2011) and different types of violence to those of Canadian-born women, thus expanding the *range of violence* that Kelly’s continuum encompasses and adding to the foundation of developing a continuum to “embrace intersectionality” (Kelly, 2012, p. xx). Consequently, we have claimed that the definition of what constitutes violence needs to be broadened beyond the physical forms of violence, as such expansion could lead to challenging the public understanding of what GBV is and to showing how “normal” and subtle (i.e., non-physical) acts of violence are treated as acceptable (Muehlenhard and Kimes, 1999).

Furthermore, our synthesis of Kelly’s theory with a feminist intersectional perspective in the case of GBV and MRW survivors allows us to understand the amplified and / or unique forms of GBV that MRW are victimized by (e.g., threats of deportation). Through the analysis of our empirical data, we have shown the implications of gendered and intersectional discriminations for increasing vulnerabilities and compounding the GBV experiences of MRW in Canada. By doing so, we have ultimately demonstrated that the *continuum is incomplete without taking into consideration all women’s social identities* (i.e., intersecting axes of oppression; Hill Collins, 1990, 2010).

Naming and comprehending the specific forms of and vulnerabilities to GBV of MRW, rather than lumping them together under GBV, is important theoretically and practically. Theoretically, it allows us to *acknowledge the differential impact of GBV to this particular population*. It is the acknowledgement of such difference that makes it possible to act consequently. From policy and program delivery perspectives, it is only through such an understanding that *targeted policies can be designed and implemented to address such violence and accomplish equity, inclusivity and citizenship* for MRW survivors of GBV.

There are three main limitations of the study. First, our study findings reflect prior literature: we know the most about IPV and least about GBV in public spaces directed at MRW. Namely, the key informants in our study spoke the most about (cis-) immigrant and refugee women victimized by male intimate partners, limiting much of our understanding of GBV against MRW to IPV. Secondly, the study is necessarily limited to the perspectives of Key Informants (e.g., service providers who work with MRW). Finally, future research should parse out the broad category of MRW and identify the specific experiences of victimized MRW by category.

## Data Availability

The de-identified raw data supporting the conclusions of this article can be provided upon a revision to and clearance from the Research Ethics Board. Requests to access the datasets should be directed to the corresponding author.

## Footnotes

^1^Kelly (1987) writes that death is an exception to the hierarchy of sexual violence consequences. Though she does not specify why, it is presumed because it is one that ends a woman’s life.

^2^The Canadian GBV program (https://www.smu.ca/gendernet/welcome.html) includes researchers at four institutions (i) Saint Mary’s University, (ii) University of New Brunswick, (iii) University of Guelph, and (iv) University of Manitoba. The research program has been approved by the Research Ethics Board at each of the four institutions. The Canadian program, funded by Canadian Institutes of Health Research, is part of the international project on *Violence Against Women Migrants and Refugees: Analyzing Causes and Effective Policy Response (GBV-MIG),* a winning project of the Gender-Net Plus Consortium (https://gbvmigration.cnrs.fr/)

^3^Interview excerpts have been edited to remove any identifying details. During analysis, we assigned each participant a number. In this article, we attribute interview excerpts using the following: KI refers to *Key Informant* followed by the corresponding participant number.

^4^Quotations have been “cleaned” for non-lexical sounds and repetition.

## References

[ref1] Abraham M. Tastsoglou C. (2016). Addressing domestic violence in Canada and the United States: the uneasy co-habitation of women and the state. Curr. Sociol. 64, 568–585. doi: 10.1177/0011392116639221

[ref2] Akers C. Kaukinen C. (2009). The police reporting behavior of intimate partner violence victims. J. Fam. Violence 24, 159–171. doi: 10.1007/s10896-008-9213-4

[ref3] Anis M. Konanur S. Mattoo D. (2013). Who—if—when to marry: The incidence of forced marriage in Ontario. Toronto, ON: South Asian Women’s Centre. Available online at: http://www.sawc.org/wp-content/uploads/2013/01/SALCO-Who-If-When-to-Marry-The-Incidence-of-Forced-Marriage-in-Ontario-Sep-2013.pdf (Accessed December 16, 2019).

[ref4] Anitha S. Gill A. (2017). “Coercion, consent and the forced marriage debate in the UK” in Marital Rights. ed. Leckey R. (New York: Routledge), 133–152.

[ref5] Barata P. C. McNally M. J. Sales I. Stewart D. E. (2005). Portuguese-speaking women voice their opinions: using their words to teach about wife abuse. Womens Health Issues 15, 134–144. doi: 10.1177/0886260505278290 15894199

[ref6] Brandl B. (2000). Power and control: understanding domestic abuse in later life. Generations 24, 39–45.

[ref7] Braun V. Clarke V. (2006). Using thematic analysis in psychology. Qual. Res. Psychol. 3, 77–101. doi: 10.1191/1478088706qp063oa

[ref8] Brennan S. (2011). “Self-reported spousal violence, 2009” in Family violence in Canada: A statistical profile. Ottawa, ON: Statistics Canada catalogue no. 85-224-X.

[ref9] Brownridge D. A. Halli S. S. (2003). Double advantage? Violence against Canadian migrant women from ‘developed’ nations. Int. Migr. 41, 29–46. doi: 10.1111/1468-2435.00229

[ref10] Carbado D. W. Crenshaw K. W. Mays V. M. Tomlinson B. (2013). Intersectionality: mapping the movements of a theory. Du Bois Rev. 10, 303–312. doi: 10.1017/S1742058X13000349, PMID: 25285150 PMC4181947

[ref11] Carman T. Elash A. (2018). “Gender persecution the top reason women seek asylum in Canada.” Canadian Broadcasting Corporation. Available online at: https://www.cbc.ca/news/canada/asylum-seekers-data-gender-persecution-1.4506245 (Accessed February 7, 2018).

[ref12] Choo H. Y. Ferree M. M. (2010). Practicing intersectionality in sociological research: A critical analysis of inclusions, interactions, and institutions in the study of inequalities. Sociol. Theory 28, 129–149.

[ref13] Côté A. Kérisit M. Côté M. L. (2001). Sponsorship--for better or for worse: The impact of sponsorship on the equality rights of immigrant women. Status of Women Canada, Ottawa, ON.

[ref14] Cotter A. (2021a). Intimate partner violence in Canada, 2018: Experiences of visible minority women in Canada. Ottawa, ON: Statistics Canada. Available online at: https://www150.statcan.gc.ca/n1/pub/85-002-x/2021001/article/00008-eng.htm (Accessed August 24, 2021).

[ref56] Cotter A. (2021b). Intimate partner violence in Canada, 2018: An overview. Ottawa, ON: Statistics Canada. Available online at: https://www150.statcan.gc.ca/n1/pub/85-002-x/2021001/article/00003-eng.htm (Accessed November 23, 2023).

[ref15] Crenshaw K. (1989). “Demarginalizing the intersection of race and sex: A black feminist critique of antidiscrimination doctrine, feminist theory and antiracist politics.” The University of Chicago Legal Forum, 139–168. https://chicagounbound.uchicago.edu/uclf/vol1989/iss1/8/ (Accessed September 10, 2024).

[ref16] Dawson M. Sutton D. Jaffe P. Straatman A. L. Poon J. Gosse M.. (2018). One is too many: Trends and patterns in domestic homicides in Canada 2010–2015. London, ON: Western University. Available online at: http://www.cdhpi.ca/ (Accessed December 6, 2018).

[ref17] Dawson M. Sutton D. Zecha A. Boyd C. Johnson A. Mitchell A. (2021). #CallItFemicide: Understanding sex/gender-related killings of women and girls in Canada 2020. Canadian Femicide Observatory for Justice and Accountability, Guelph, ON. Available online at: https://femicideincanada.ca/callitfemicide2020.pdf (Accessed April 13, 2021).

[ref18] Dearham K. K. (2017). “We just know who we are”: lesbian refugees in the Canadian immigration system. Masters thesis. Toronto, ON: York University. Available online at: https://yorkspace.library.yorku.ca/xmlui/bitstream/handle/10315/34108/KDearham%20PRP.pdf?sequence=1 (Accessed September 10, 2024).

[ref19] DeKeseredy W. S. Schwartz M. D. (2001). “Definitional issues” in Sourcebook on Violence against Women. eds. Renzetti C. M. Edleson J. L. Bergen R. K. (Thousand Oaks, CA: Sage Publications, Inc.), 23–34.

[ref20] Dunham K. Senn C. Y. (2000). Minimizing negative experiences. J. Interpers. Violence 15, 251–261. doi: 10.1177/088626000015003002

[ref21] Duron J. F. Johnson L. Hoge G. L. Postmus J. L. (2021). Observing coercive control beyond intimate partner violence: examining the perceptions of professionals about common tactics used in victimization. Psychol. Violence 11, 144–154. doi: 10.1037/vio0000354

[ref22] Erez E. Adelman M. Gregory C. (2009). Intersections of immigration and domestic violence: voices of battered immigrant women. Fem. Criminol. 4, 32–56. doi: 10.1177/1557085108325413

[ref23] Erez E. Harper S. (2018). “Intersectionality, immigration, and domestic violence” in The Handbook of Race, Ethnicity, Crime, and Justice. eds. Martínez R. Jr. Hollis M. E. Stowell J. I. (Hoboken, NJ: John Wiley & Sons, Inc.), 457–474.

[ref24] Eriksson M. Ulmestig R. (2021). ‘It’s not all about money’: toward a more comprehensive understanding of financial abuse in the context of VAW. J. Interpers. Violence 36:NP1625-1651NP. doi: 10.1177/0886260517743547 29295038

[ref25] Fitzgerald A. J. Barrett B. J. Stevenson R. Cheung C. H. (2019). Animal maltreatment in the context of intimate partner violence: a manifestation of power and control? Violence Against Women 25, 1806–1828. doi: 10.1177/1077801218824993, PMID: 30714886

[ref26] Fox S. D. Griffin R. H. Pachankis J. E. (2020). Minority stress, social integration, and the mental health needs of LGBTQ asylum seekers in North America. Soc. Sci. Med. 246, 1–10. doi: 10.1016/j.socscimed.2019.112727, PMID: 31881451

[ref27] Government of Canada (2021). Gender-based analysis plus. Ottawa, ON. Available online at: https://www.canada.ca/en/immigration-refugees-citizenship/corporate/publications-manuals/departmental-performance-reports/2020/gender-based-analysis-plus.html (Accessed September 10, 2024).

[ref28] Hill Collins P. (2010). The new politics of community. Am. Sociol. Rev. 75, 7–30. doi: 10.1177/0003122410363293

[ref29] Hill Collins P. (1990). Black Feminist Thought: Knowledge, Consciousness, and the Politics of Empowerment. Boston: Unwin Hyman.

[ref30] Hill Collins P. (2015). Intersectionality's definitional dilemmas. Annu. Rev. Sociol. 41, 1–20. doi: 10.1146/annurev-soc-073014-112142

[ref31] Holtmann C. Tastsoglou E. Dawson M. Wilkinson L. (2023). Surviving gender-based violence: A social ecological approach to migrant women’s resilience. Can. Ethn. Stud. 55, 57–77. doi: 10.1353/ces.2023.a928884

[ref32] Hudon T. (2015). Immigrant women in women in Canada: A gender-based statistical report. Ottawa, ON: Statistics Canada. Available online at: https://www150.statcan.gc.ca/n1/en/pub/89-503-x/2015001/article/14217-eng.pdf?st=YC6cBXUw (Accessed September 10, 2024).

[ref33] Ibrahim D. (2018). “Trafficking in persons in Canada, 2016” in *Canadian Centre for Justice Statistics*. Catalogue no. 85-005-X, Ottawa, ON. Available online at: https://www150.statcan.gc.ca/n1/en/pub/85-005-x/2018001/article/54979-eng.pdf?st=wFeeYV7p (Accessed September 11, 2024).

[ref34] Jiwani Y. (2011). Discourses of Denial: Mediations of Race, Gender, and Violence. Vancouver, BC: UBC Press.

[ref35] Kaye J. Winterdyk J. Quarterman L. (2014). Beyond criminal justice: A case study of responding to human trafficking in Canada. Can. J. Criminol. Crim. Justice 56, 23–48. doi: 10.3138/cjccj.2012.E33

[ref1002] Kelly L. (1987). “The Continuum of Sexual Violence” in Women, Violence and Social Control. eds. Hanmer J. Maynard M. (London: Palgrave Macmillan), 46–60. doi: 10.1007/978-1-349-18592-4_4

[ref36] Kelly L. (2012). “Standing the test of time? Reflections on the concept of the continuum of sexual violence” in Handbook on Sexual Violence. eds. Brown J. M. Walklate S. L. (Oxfordshire, UK: Routledge), xvii–xxvi.

[ref37] Kim C. (2019). Social isolation, acculturative stress and intimate partner violence (IPV) victimization among Korean immigrant women. Int. J. Intercult. Relat. 72, 87–95. doi: 10.1016/j.ijintrel.2019.07.005

[ref38] Korteweg A. C. Yurdakul G. (2012). Religion, culture and the politicization of honour-related violence: A critical analysis of media and policy debates in Western Europe and North America. Geneva, CH: UN Research Institute for Social Development. Available online at: http://www.unrisd.org/unrisd/website/document.nsf/ (Accessed June 20, 2011).

[ref39] Krause U. (2015). A continuum of violence? Linking sexual and gender-based violence during conflict, flight, and encampment. Refug. Surv. Q. 34, 1–19. doi: 10.1093/rsq/hdv014

[ref40] Krebs C. Breiding M. J. Browne A. Warner T. (2011). The association between different types of intimate partner violence experienced by women. J. Fam. Violence 26, 487–500. doi: 10.1007/s10896-011-9383-3

[ref41] Lister R. (1998). Citizenship and difference: towards a differentiated universalism. Eur. J. Soc. Theory 1, 71–90. doi: 10.1177/136843198001001006

[ref42] Lockyer S. Savigny H. (2020). Rape jokes aren’t funny: the mainstreaming of rape jokes in contemporary newspaper discourse. Fem. Media Stud. 20, 434–449. doi: 10.1080/14680777.2019.1577285

[ref43] Lokot M. (2021). Whose voices? Whose knowledge? A feminist analysis of the value of key informant interviews. Int J Qual Methods 20:160940692094877. doi: 10.1177/1609406920948775

[ref44] Marfelt M. M. (2016). Grounded intersectionality: key tensions, a methodological framework, and implications for diversity research. Equal. Divers. Inclus. 35, 31–47. doi: 10.1108/EDI-05-2014-0034

[ref45] Matheson F. I. Daoud N. Hamilton-Wright S. Borenstein H. Pedersen C. O’Campo P. (2015). Where did she go? The transformation of self-esteem, self-identity, and mental well-being among women who have experienced intimate Ppartner violence. Womens Health Issues 25, 561–569. doi: 10.1016/j.whi.2015.04.006, PMID: 26116987

[ref46] Muehlenhard C. L. Kimes L. A. (1999). The social construction of violence: the case of sexual and domestic violence. Personal. Soc. Psychol. Rev. 3, 234–245. doi: 10.1207/s15327957pspr0303_6, PMID: 15661674

[ref47] Murphy C. M. O’Leary K. D. (1989). Psychological aggression predicts physical aggression in early marriage. J. Consult. Clin. Psychol. 57, 579–582. doi: 10.1037/0022-006X.57.5.579, PMID: 2794178

[ref48] Oliveira C. Martins M. D. R. O. Dias S. Keygnaert I. (2019). Conceptualizing sexual and gender-based violence in European asylum reception centers. Arch. Public Health 77, 1–11. 31164983 10.1186/s13690-019-0351-3 PMC6545000

[ref49] Phillimore J. Pertek S. Akyuz S. Darkall H. Hourani J. McKnight P.. (2022). ‘We are forgotten’: forced migration, sexual and gender-based violence, and coronavirus Disease-2019. Violence Against Women 28, 2204–2230. doi: 10.1177/10778012221079374 34533382 PMC9118490

[ref51] Powell A. Henry N. (2018). Policing technology-facilitated sexual violence against adult victims: police and service sector perspectives. Polic. Soc. 28, 291–307. doi: 10.1080/10439463.2016.1154964

[ref52] Reilly N. Bjørnholt M. Tastsoglou E. (2022). “Vulnerability, Precarity and intersectionality: A critical review of three key concepts for understanding gender-based violence in migration contexts” in Gender-Based Violence in Migration: Interdisciplinary, Feminist and Intersectional Approaches. eds. Freedman J. Sahraoui N. Tastsoglou E. (London, UK: Palgrave-Macmillan), 29–56.

[ref53] Sahraoui N. Freedman J. (2022). “Gender-based violeence as a continuum in the lives of women seeking asylum: from resistance to patriarchy to patterns of institutional violence in France” in Gender-Based Violence in Migration: Interdisciplinary, Feminist and Intersectional Approaches. eds. Freedman J. Sahraoui N. Tastsoglou E. (London, UK: Palgrave-Macmillan), 211–234.

[ref54] Senn C. Y. (2000). “Violence” in Encyclopedia of Feminist Theories. ed. Code L. (London, UK: Routledge), 482–483.

[ref55] Sev’er A. (1997). Recent or imminent separation and intimate violence against women: A conceptual overview and some Canadian examples. Violence Against Women 3, 566–589. doi: 10.1177/1077801297003006002 12295554

[ref57] Statistics Canada (2023). Table 98-10-0347-01 immigrant status and period of immigration by gender and age: Canada, provinces and territories. Ottawa, ON. Available online at: https://www150.statcan.gc.ca/t1/tbl1/en/tv.action?pid=9810034701&pickMembers%5B0%5D=2.1&pickMembers%5B1%5D=3.3&pickMembers%5B2%5D=4.1 (Accessed 20 July 2024).

[ref58] Sullivan C. Block K. Vaughan C. (2021). “The continuum of gender-based violence across the refugee experience” in Understanding Gender-Based Violence: An Essential Textbook for Nurses, Healthcare Professionals and Social Workers. eds. Bradbury-Jones C. Isham L. (Cham, CH: Springer), 33–47.

[ref59] Sullivan T. P. McPartland T. S. Armeli S. Jaquier V. Tennen H. (2012). Is it the exception or the rule? Daily co-occurrence of physical, sexual, and psychological partner violence in a 90-day study of substance-using, community women. Psychol. Violence 2, 154–164. doi: 10.1037/a0027106, PMID: 24349863 PMC3859524

[ref60] Tastsoglou E. (2019). Transnational, feminist and intersectional perspectives on immigrants and refugees in Canada: an introduction. Can. Ethn. Stud. 51, 1–16. doi: 10.1353/ces.2019.0017

[ref61] Tastsoglou E. (2022). “Gender and the continuum of violence in forced migration: asylum-seeking women in the Eastern Mediterranean” in An International Workshop on “Gendering Violence and Precarity in Forced Migration: Asylum Seeking Women in the Eastern Mediterranean (WASEM)” Halifax, NS. Available online at: https://www.smu.ca/gendernet/related-projects.html, 3. (Accessed July 20, 2024).

[ref62] Tastsoglou E. Nourpanah S. (2019). (re)producing gender: refugee advocacy and sexual and gender-based violence in refugee narratives. Can. Ethn. Stud. 51, 37–56. doi: 10.1353/ces.2019.0019

[ref63] Thompson R. S. Bonomi A. E. Anderson M. Reid R. J. Dimer J. A. Carrell D.. (2006). Intimate partner violence: prevalence, types, and chronicity in adult women. Am. J. Prev. Med. 30, 447–457. doi: 10.1016/j.amepre.2006.01.016 16704937

[ref64] Toews M. L. Bermea A. M. (2017). “I was naive in thinking, ‘I divorced this man, he is out of my life’”: A qualitative exploration of post-separation power and control tactics experienced by women. J. Interpers. Violence 32, 2166–2189. doi: 10.1177/0886260515591278, PMID: 26088900

[ref66] Velonis A. J. Daoud N. Matheson F. Woodhall-Melnik J. Hamilton-Wright S. O’Campo P. (2017). Strategizing safety: theoretical frameworks to understand Women’s decision making in the face of partner violence and social inequities. J. Interpers. Violence 32, 3321–3345. doi: 10.1177/0886260515598953, PMID: 26303937

[ref67] Werbner P. Yuval-Davis N. (1999). “Introduction: women and the new discourse of citizenship” in Women, Citizenship and Difference. eds. Yuval-Davis N. Werbner P. (London, New York: Zed Books), 1–38.

